# EFFICACY OF KINESIOTAPING DURING REHABILITATION FOLLOWING TOTAL KNEE ARTHROPLASTY: A PROSPECTIVE RANDOMIZED CONTROLLED TRIAL

**DOI:** 10.2340/jrm.v58.44964

**Published:** 2026-07-17

**Authors:** Ahmed AL-SAADI, Bernd WEGENER, Andreas VEIHELMANN

**Affiliations:** 1 SRH Health Centre, Bad Herrenalb; 2 Department of Orthopaedics, Trauma Surgery and Rehabilitative Medicine, Ludwig-Maximilians-University of Munich, Munich; 3 Sports Clinic, Klinikum Stuttgart, Stuttgart, Germany

**Keywords:** kinesiotaping, total knee arthroplasty, rehabilitation, knee joint function, pain relief

## Abstract

**Objective:**

To determine whether adding kinesiotaping to standard inpatient rehabilitation reduces early postoperative swelling and improves function after total knee arthroplasty.

**Design:**

Prospective, randomized, controlled clinical trial.

**Patients:**

136 randomized post-total knee arthroplasty inpatients; 102 completed (kinesiotaping *n* = 51; control *n* = 51).

**Methods:**

Both groups received a standardized 3-week programme. Kinesiotaping was applied by a trained physician following K-Active protocols from postoperative day 10 to day 25. The primary outcome was change in knee circumference (cm) from day 10 to day 25. Secondary outcomes were numeric rating scale pain, passive range of motion, and functional tests (Timed Up and Go, chair stand, 10-m walk).

**Results:**

Swelling reduction was greater with kinesiotaping: mean change 2.1 (SD 1.7) cm vs 0.9 (1.4) cm; mean difference 1.2 cm (95% CI 0.7–1.7), *p* < 0.001. Flexion gain was larger with kinesiotaping: 18.3° (7.7°) vs 14.4° (10.0°); mean difference 3.9° (95% CI 0.4–7.4), *p* = 0.03. Pain reduction and other secondary outcomes did not differ significantly. No kinesiotaping-related adverse events occurred.

**Conclusion:**

Kinesiotaping added to standard rehabilitation reduced early postoperative knee swelling and produced a small improvement in flexion after total knee arthroplasty. Further work should examine durability and patient-centred outcomes.

**Trial registration:**

*ClinicalTrials.gov* Identifier: NCT06831682.

**Registration date:**

February 8, 2025.

## LAY ABSTRACT

After knee replacement, patients often have pain, swelling, and limited movement. We tested whether adding elastic kinesiotaping to standard inpatient rehabilitation could improve recovery during the first weeks. In our randomized study, all patients followed the same rehabilitation plan. Half also received kinesiotaping on the operated knee from day 10 to day 25 after surgery. Compared with patients without kinesiotaping, those with kinesiotaping had a larger reduction in knee swelling and a small extra gain in knee bending. Pain and walking tests were similar. No tape-related skin problems occurred. These results suggest that kinesiotaping can safely reduce early swelling after knee replacement and may slightly improve bending, but its effects on pain and everyday function still need study.

Total knee arthroplasty (TKA) surgeries rank among the most frequently performed elective procedures in orthopaedics and trauma surgery globally. In Germany, approximately 165,000 TKAs are implanted annually. Germany holds the third position worldwide in the number of TKA surgeries, with a rate of 207 procedures per 100,000 inhabitants. While the majority of patients are between 55 and 84 years old, a growing proportion of younger patients are under the age of 55 (1–3).

Inpatient rehabilitation is a key component of recovery in Germany, particularly after TKA. Early initiation of structured physiotherapy, including exercises for range of motion (ROM), quadriceps strengthening, gait training, and daily activities, is essential, alongside effective pain management. Supervised post-discharge exercise programmes yield better short-term functional outcomes than standard care, although inpatient rehabilitation has no significant advantage over home-based therapy (4, 5).

Aquatic therapy, in addition, has proven effective in early postoperative rehabilitation, enhancing strength and ROM more significantly than home exercises (5, 6). However, manual lymphatic drainage has shown limited efficacy in reducing swelling or accelerating recovery after TKA, although it may offer short-term pain relief (7, 8). These findings highlight the importance of evidence-based protocols in rehabilitation programmes in Germany.

Kinesiotaping (KT) is commonly applied in postoperative rehabilitation because of its purported benefits in normalizing muscle function, enhancing lymphatic and blood flow, alleviating pain, and correcting joint misalignments.

It is estimated that KT elevates the skin, thereby increasing blood circulation and lymphatic flow, reducing inflammation, and promoting injury healing. It also alleviates pain, regulates muscle and nerve tension, and improves joint function. Radiological assessments have demonstrated mechanical changes in the tissue beneath KT.

KT involves 6 distinct techniques, each requiring specialized knowledge and precise application to ensure therapeutic efficacy. The muscle technique, which is commonly used, supports or relaxes muscle function by applying unstretched tape to the skin, typically away from tendons unless pain necessitates otherwise. Ligament taping employs full tension in the tape’s centre to activate mechanoreceptors, whereas fascial taping, which uses a V-shaped application with moderate tension, corrects fascia alignment and alleviates movement-related pain (9).

Additional methods address functional and physiological needs. The correction technique enhances tissue elasticity and alignment with tape stretched to 50–70% of its length, facilitating improved mobility. Lymphatic drainage taping reduces swelling and optimizes lymph circulation through minimal tension applied in specific patterns, whereas functional movement taping supports mobility in cases of muscle dysfunction via moderate-to-high tension (9). These techniques highlight the adaptability of KT in tailored rehabilitation, particularly post-surgery.

Evidence regarding the effectiveness of KT after TKA remains limited and mixed, particularly in the early postoperative rehabilitation phase. Therefore, it is of interest to explore whether KT has positive effects on patients in postoperative rehabilitation after TKA surgeries and whether the effort for its routine postoperative application is justified. All these effects are desirable for postoperative treatment following TKA.

We hypothesized that the application of KT during early postoperative rehabilitation after TKA would lead to greater reductions in knee swelling and improved knee ROM than would standard rehabilitation alone. A power calculation was conducted to ensure that the study was adequately powered to detect a clinically meaningful difference in knee swelling (see Statistical Analysis). The aim of this study was to investigate whether the application of KT in postoperative rehabilitation for TKA patients, compared with TKA patients without the application of KT, promotes an earlier postoperative outcome by reducing swelling and pain in the surgical area and improving muscle strength and thus knee joint function.

## PATIENTS AND METHODS

### Study design and ethics

This prospective, randomized, controlled clinical study evaluated the efficacy of KT in patients who underwent primary TKA and were admitted for inpatient rehabilitation at SRH Health Centre Bad Herrenalb.

The study began in March 2023 and lasted 1.5 years. The protocol was approved by the Ethics Committee of the Baden-Württemberg Medical Association (Landesärztekammer Baden-Württemberg, reference F-2021-085).

The trial was registered at ClinicalTrials.gov (NCT06831682) on 8 February 2025. Registration occurred after the start of patient enrolment and was therefore retrospective. This is acknowledged as a limitation of the study. The study protocol, eligibility criteria, intervention procedures, and primary outcome were defined before data analysis.

### Participants

Eligible patients were adults (≥18 years) who had undergone primary TKA with uncomplicated wound healing and no signs of infection.

Exclusion criteria were revision surgery, early postoperative complications (e.g., wound healing disorders, infection, thrombosis), severe comorbidities (e.g., NYHA class III–IV heart failure, stage 3–4 renal insufficiency, chronic lymphedema, or dermatological disease), adhesive allergy, neurological or neuromuscular disorders, and cognitive or compliance limitations.

A total of 421 patients were screened; 136 met the inclusion criteria and were randomized into 2 groups receiving identical rehabilitation and analgesic protocols. Group 1 received KT in addition to standard rehabilitation; Group 2 underwent standard rehabilitation only. Ultimately, 102 participants (51 per group) completed the entire study.

All participants underwent primary TKA. Detailed comparative analyses of implant design and operative technique were not prespecified and were not available in a sufficiently standardized form for robust subgroup analysis.

### Randomization and allocation concealment

Participants were randomized 1:1 to KT + standard rehabilitation or standard rehabilitation alone using a computer-generated permuted-block sequence in Microsoft Excel (block sizes 4 and 6) (Microsoft Corp, Redmond, WA, USA). Allocation was revealed sequentially just before the first taping or control session.

The same physician applied the intervention and performed all outcome assessments; blinding was therefore not possible, but potential bias was reduced by standardized measurement procedures, fixed anatomical landmarks, and objective instruments.

Sex was recorded as a baseline characteristic; however, randomization was not stratified by sex. Because the study was powered for the primary endpoint in the overall cohort rather than for subgroup comparisons, sex-specific analyses were considered exploratory.

### Rehabilitation protocol

Both groups participated in the same standardized 3-week inpatient rehabilitation programme. The programme included routine physiotherapy 3 times weekly, aquatic therapy 4 times weekly, manual lymphatic drainage 3 times weekly, gait training daily, and supervised therapeutic exercises according to institutional practice for post TKA rehabilitation.

Each treatment session lasted approximately 45 min. Physiotherapy focused on pain-adapted mobilization, restoration of knee flexion and extension, quadriceps activation, transfer training, gait training, and stair practice when appropriate. The content and progression of therapy were standardized at the institutional level, although minor individual adjustments were allowed according to patient tolerance and daily clinical status.

Analgesic treatment followed the usual institutional postoperative regimen and did not differ systematically between groups.

As rehabilitation was delivered in an inpatient setting, attendance at scheduled therapy sessions was monitored clinically and overall compliance was considered high. However, formal session by session adherence data were not prospectively recorded for statistical analysis.

### Kinesiotaping procedure

KT was applied by a physician trained and certified according to the official K-Active Europe GmbH (Minden, Germany) protocol, using lymphatic, muscle/fascial, and ligament techniques.

Lymphatic taping followed a fan-shaped configuration originating from the mid-thigh towards the peripatellar area; muscle/fascial strips were applied along the quadriceps with 25–35% tension and replaced every 3 days. Each session lasted 15 min. Applications were performed with the patient supine and relaxed and renewed at regular intervals until postoperative day 25. A representative illustration of KT placement has been added as Fig. S1.

### Outcome measures

Outcome assessments were performed at 2 predefined time points: postoperative day 10 and postoperative day 25. This time window was chosen because wound conditions were sufficiently stable for KT application while clinically relevant postoperative swelling and mobility limitation were still present.

The primary outcome was the change in knee joint circumference (cm) from postoperative day 10 to day 25, measured with a non-elastic tape at a fixed anatomical landmark 2 cm proximal to the superior patellar border in the supine position.

To enhance repeatability, the assessor performed 2 measurements per time point and used the mean value. All measurements were performed by the same physician to minimize inter-observer variability.

We did not formally quantify intra-rater repeatability; however, a single trained assessor and standardized landmarking were used to reduce error.

Passive ROM was measured using a manual goniometer in the supine position, with the hip neutral and the heel supported to assess extension lag. Calibration and positioning were checked regularly to ensure consistency.

Secondary outcomes included pain NRS, and functional performance (Timed Up and Go, chair stand, and 10-m walk tests). Each test was performed under identical conditions for both groups.

These measures were chosen to capture complementary domains of early postoperative recovery, namely oedema, pain, knee mobility, transfer performance, and walking ability.

Adverse events (AEs) were actively monitored at each assessment. At every visit, patients were specifically asked about skin reactions (itching, erythema, blistering, or breakdown), allergy, and tape-related discomfort. No KT-related AEs were observed; specifically, zero skin reactions were reported in the KT group.

### Psychometric treatment of scales and multiplicity

NRS and functional tests include ordinal elements; as per JRM guidance we report medians (quartiles) for ordinal variables and means (95% CIs) for interval data. There was 1 prespecified primary endpoint (no multiplicity adjustment). Secondary endpoints were treated as exploratory with 95% CIs.

### Statistical analysis

*A priori* calculations (G-Power, two-sample *t*-test, two-tailed) targeted 80% power at α = 0.05 to detect a standardized mean difference (Cohen’s *d* = 0.5) in the primary outcome (change in knee circumference from postoperative day 10 to day 25). This required *n* = 102 (51 per group). Anticipating attrition, we randomized *n* = 136, which yielded 51 per group completing the primary outcome assessment.

The calculation assumed a between-group mean difference of 0.8 cm in change of knee circumference (day 10→25) with a common SD of 1.6 cm, corresponding to Cohen’s *d* = 0.50, two-sided α = 0.05, and 80% power using a two-sample *t*-test. This required *n* = 102 analysable participants (51 per group). We planned for ~25% attrition; therefore, we aimed to randomize *n*≈136. In practice, 136 participants were randomized and 102 completed the primary endpoint, matching the target.

The primary endpoint was the between-group difference in knee circumference change (cm) from day 10 to day 25. Secondary endpoints (exploratory) included pain (NRS), passive knee ROM (flexion, extension), and functional tests (Timed Up and Go, chair stand, and 10-m walk).

Between-group comparisons used *t*-tests. We report mean differences with 95% confidence intervals and *p*-values. Because there was 1 prespecified primary endpoint, no multiplicity adjustment was applied; secondary outcomes are exploratory, and 95% CIs are provided.

Sensitivity analyses using nonparametric tests yielded similar results. All analyses were conducted using SPSS v23 (IBM Corp, Armonk, NY, USA).

## RESULTS

The 2 study groups (KT and control) were comparable in key baseline characteristics, including age, sex, and operated knee side. The slight difference in average age between the KT group and the control group does not suggest a significant influence on the primary outcomes. Additionally, the sex distribution and the predominance of left-knee surgeries in both groups confirmed that the baseline characteristics were well matched between the 2 study arms (Table I).

**Table I:** Baseline characteristics of the participants by group

Characteristic	Control (*n* = 51)	KT (*n* = 51)	Total (*n* = 102)
Mean age (SD)	70.4 (± 7.5)	73.4 (± 6.9)	71.9 (± 7.2)
Gender			
Male (%)	19 (37.3%)	24 (47.1%)	43 (42.2%)
Female (%)	32 (62.7%)	27 (52.9%)	59 (57.8%)
Side of knee surgery			
Left knee (%)	32 (62.7%)	27 (52.9%)	59 (57.8%)
Right knee (%)	19 (37.3%)	24 (47.1%)	43 (42.2%)

KT: kinesiotaping.

**Fig. 1:**
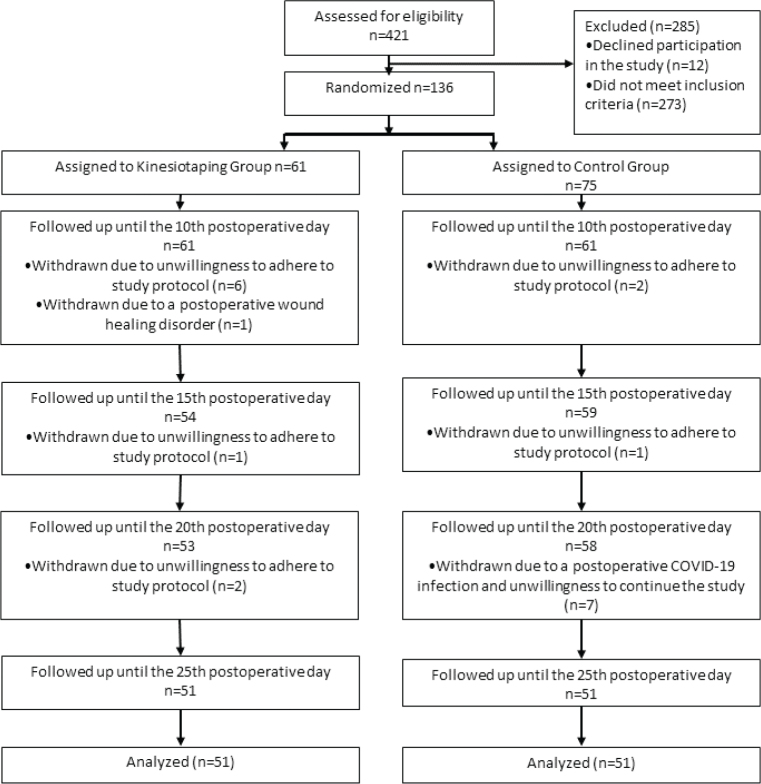
Study flowchart.

The flowchart (Fig. 1) illustrates the progression of participants throughout the study. The flow diagram follows CONSORT 2010 guidelines and shows the number of patients screened, excluded with reasons, randomized, allocated, followed up, and analysed. A total of 421 potential participants were screened, of whom 136 were randomized after a thorough evaluation of the inclusion criteria. The participants were evenly allocated to an intervention group (KT) or a control group (without KT). During the 25-day postoperative follow-up period, significant dropout occurred in both groups, which was mainly due to patient refusal, minor postoperative complications, or COVID-19 infection. These variables were evenly distributed between groups and did not significantly affect group comparability.

Patients with major postsurgical complications were excluded during screening or the early postoperative period. Among the initially randomized cohort, minor complications (e.g., delayed wound healing) occurred in both groups, with no significant difference in frequency or type. No adverse effects related to KT were observed. There were no reports of allergic reactions, skin irritation, or discomfort due to the tape throughout the intervention period.

Additional reasons for withdrawal included postoperative complications, such as wound healing disorders, and external factors, such as COVID-19 infection. Despite these dropouts, the study was completed with a comparable number of 51 participants per group, enabling a direct comparison of the primary and secondary outcomes between the two intervention groups.

Table II presents a statistical comparison of the postoperative parameters between patients who received KT and those who did not during rehabilitation following TKA. It highlights key outcomes such as pain reduction, knee circumference reduction (swelling), and ROM (flexion) and results from functional tests.

**Table II:** Statistical analysis of postoperative parameters between patients with kinesiotaping (KT) and control group

Test	Group	*n*	Mean	SD	SEM	*p*-value
Pain reduction (NRS)	Control	51	1.6	1.7	0.2	0.06
	KT	51	2.2	1.8	0.2	
Knee circumference (swelling reduction)	Control	51	0.9	1.4	0.1	0.00
	KT	51	2.1	1.7	0.2	
ROM (flexion)	Control	51	14.4	10.0	1.4	0.03
	KT	51	18.3	7.7	1.0	
Timed Up and Go test	Control	51	4.6	3.6	0.5	0.37
	KT	51	5.2	3.2	0.4	
Chair stand test	Control	51	4.5	4.6	0.6	0.27
	KT	51	5.5	4.6	0.6	
10-m walk test	Control	51	4.0	4.4	0.6	0.57
	KT	51	3.7	2.1	0.3	

SD: standard deviation; SEM: standard error of mean; NRS: numeric rating scale; ROM: range of motion.

The comparison of pain reduction as measured by the NRS between the 10th and 25th postoperative days revealed that the KT group had a greater reduction in pain (2.2 points) than did the control group (1.6 points). However, this difference was not statistically significant (*p*-value = 0.06). Both groups showed considerable variability in pain reduction, suggesting that KT may have a positive effect.

Mean (SD) reduction was 2.1 (1.7) cm in the KT group vs 0.9 (1.4) cm in the control group. The between-group mean difference was 1.2 cm (95% CI 0.7–1.7; *p* < 0.001), indicating a statistically and clinically significant greater reduction in swelling with KT. This effect size (Cohen’s *d*≈0.76) corresponds to a moderate to large benefit.

### ROM (flexion)

With respect to knee extension, no significant between-group difference was found (*p*-value = 0.24). However, there was a significant difference in knee flexion:

A significant between-group difference was found in knee flexion, with the KT group showing a greater increase (18.3° vs 14.4°) and a *p*-value of 0.03. These findings suggest that KT has a clinically meaningful effect on improving knee flexion post-surgery.

No statistically significant between-group differences were found for the Timed Up and Go test (*p*-value > 0.05).

KT led to a significantly greater reduction in knee swelling (*p*-value < <luee to a significantly greaterknee flexion than did the control (*p*-value = 0.03).

No significant differences were observed for pain relief, knee extension, or functional tests (Timed Up and Go, chair stand, and 10-m walk).

**Fig. 2:**
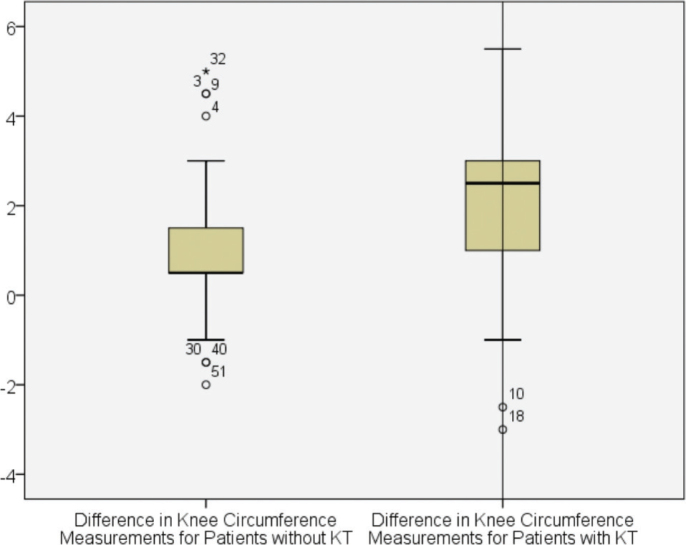
Differences in knee circumference measurements between the 2 study groups.

The box-whisker plot (Fig. 2) highlights a significant reduction in knee circumference in the KT group compared with the group without KT, with a narrower interquartile range indicating more consistent results. The KT group presented a mean reduction of 2.1 cm, which was significantly greater than the 0.9 cm reduction observed in the group without KT (*p*-value < 0.05). These findings underscore the efficacy of KT in reducing knee swelling following TKA, with more predictable outcomes in the KT group.

**Fig. 3:**
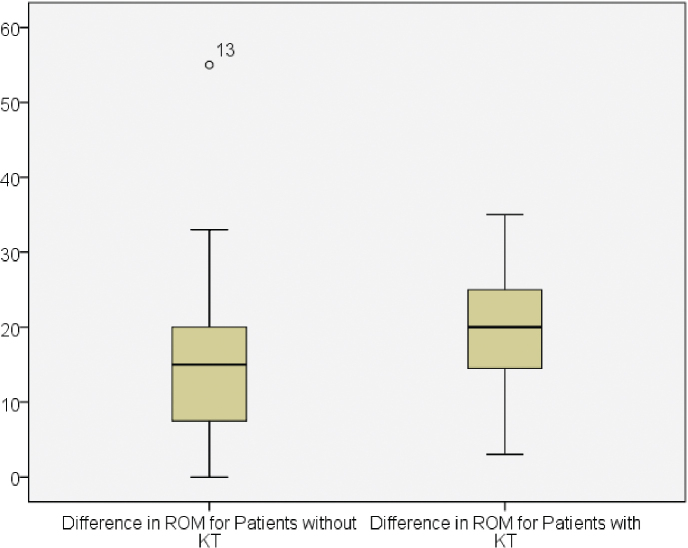
Differences in knee ROM between the 2 study groups.

Boxplot analysis from Fig. 3 revealed a statistically significant (*p*-value = 0.03) increase in knee ROM within the KT cohort compared with the control group. Specifically, the KT group presented a median elevation of 18.3°, whereas the control group presented a median increase of 14.4°. Furthermore, the KT group displayed diminished variability, suggesting more consistent outcomes. These findings provide empirical support for the assertion that KT exerts a positive influence on knee flexion following TKA, yielding a clinically meaningful effect.

## DISCUSSION

In this study, we applied a combination of lymphatic, muscle/fascial, and ligament taping, based on standardized protocols outlined by Jaron et al. (2021) (9).This approach aims to optimize postoperative outcomes by reducing swelling, improving mobility, and facilitating recovery after TKA surgery.

One of the key findings was a significant reduction in knee circumference in the KT group compared with the control group. The observed 2.1 cm reduction in knee circumference in the KT group not only reached statistical significance but also exceeded clinically meaningful thresholds reported in the literature. Previous studies have defined a minimal clinically important difference (MCID) of approximately 0.7–1.0 cm for postoperative oedema reduction following TKA (11–13). These findings support the clinical relevance of our findings and highlight the value of KT in managing early postoperative swelling. The reduction of 2.1 cm in the KT group compared with 0.9 cm in the control group suggests that KT has a clinically significant effect on reducing postoperative knee swelling, which in turn leads to faster recovery and pain reduction in these patients.

While this outcome is consistent with other studies showing KT’s potential in reducing oedema (14, 15), the possibility of bias due to differences in manual lymph drainage was ruled out, as both groups received similar amounts.

Other studies have also supported the use of KT for reducing oedema. For example, 1 study reported that lymphatic KT significantly reduced subcutaneous tissue oedema after TKA, although it had no effect on mobility (15). Additionally, a prospective randomized study revealed that KT helps reduce pain and swelling in the early phase after anterior cruciate ligament reconstruction surgery (14).

The observed reduction in knee circumference is likely due to KT, although inactivity-related muscle atrophy could have contributed. However, given the short 10–25-day postoperative period in our study, muscle atrophy, which typically takes 3–4 weeks to manifest, is unlikely to have significantly influenced the results. Thus, KT is the more probable cause of the observed improvements (16).

In our study, the KT group demonstrated a greater mean increase in knee flexion than the control group, although both groups improved over time. In the context of TKA rehabilitation, a change of ≥5° to 10° in knee flexion is often considered the minimal clinically important difference (MCID) (17, 18). In our study, the KT group showed a mean improvement of 18.3°, compared with 14.4° in the control group, resulting in a statistically significant between-group difference of approximately 4°. While this falls near the lower limit of clinical relevance, it may still have functional implications for early postoperative recovery, especially considering the non-invasive nature of KT.

Improvements in knee extension were observed in both groups. However, it is important to note that full extension is achieved primarily during surgery through implant positioning and soft tissue balancing and may be less influenced by postoperative interventions such as KT.

Our findings suggest that KT may support knee flexion and swelling reduction during early postoperative recovery following TKA. However, further research is needed to clarify its specific benefits and to explore whether any additive effects exist alongside standard physiotherapy.

Although previous studies have shown that KT may reduce pain, our study did not find a significant difference in pain relief between the KT and control groups. The small-to-moderate effect size (Cohen’s *d* = 0.4) suggests that KT could still have some clinically relevant impact on pain relief, although further research with larger samples is needed to confirm this finding.

While the literature shows mixed findings, some studies suggest that KT benefits pain reduction and mobility, but further research is needed to explore the long-term effects of KT in postoperative rehabilitation.

KT appears to be a safe and easily applicable adjunct to standard rehabilitation after TKA, with potential clinical value in reducing early postoperative swelling and modestly improving knee flexion. Given that oedema and limited mobility are key barriers to early recovery, the observed effects may facilitate participation in rehabilitation and support functional progress in the initial postoperative phase. Although no significant benefits were found for pain or functional performance, the simplicity, low cost, and absence of adverse events support its use as a complementary intervention. Future research should evaluate whether these early benefits translate into meaningful long-term improvements in patient-centred outcomes and identify subgroups most likely to benefit.

In conclusion, adding KT to standard inpatient rehabilitation after TKA significantly reduced postoperative swelling and modestly improved knee flexion, without adverse effects. These findings support KT as a safe adjunct during early rehabilitation.

### Limitations

This study has several limitations. First, the trial was not blinded, as the same physician applied the intervention and performed outcome assessments, which may introduce measurement bias despite the use of standardized procedures. Second, the KT group had a slightly greater proportion of male participants. Although this difference was not statistically significant, sex-related factors such as muscle recovery and pain perception may have influenced the outcomes; future studies should consider stratified randomization. Third, although the sample size was adequately powered for the primary outcome, it may have been insufficient to detect smaller but clinically meaningful differences in secondary outcomes. Participant dropout due to complications or withdrawal may have introduced minor bias, although group comparability was maintained. In addition, variability in rehabilitation intensity and the lack of formal adherence tracking may have influenced the results.

Furthermore, the subjective nature of pain assessment introduces the possibility of placebo effects. Knee circumference was measured only on the operated limb, without comparison with the contralateral side, limiting interpretation of swelling changes and making it difficult to fully distinguish treatment effects from natural postoperative recovery. The retrospective registration of the trial is also acknowledged as a methodological limitation. Finally, the absence of long-term follow-up prevents conclusions regarding the durability of the observed effects.

## Supplementary Material


