# Supplementary figures and images for: EFFICACY OF KINESIOTAPING DURING REHABILITATION FOLLOWING TOTAL KNEE ARTHROPLASTY: A PROSPECTIVE RANDOMIZED CONTROLLED TRIAL
Source: J Rehabil Med. 2026 Jul 17;58:44964. doi: 10.2340/jrm.v58.44964 (PMC13386228; doi:10.2340/jrm.v58.44964)

Fig. S1. A representative illustration of kinesiotaping.

1

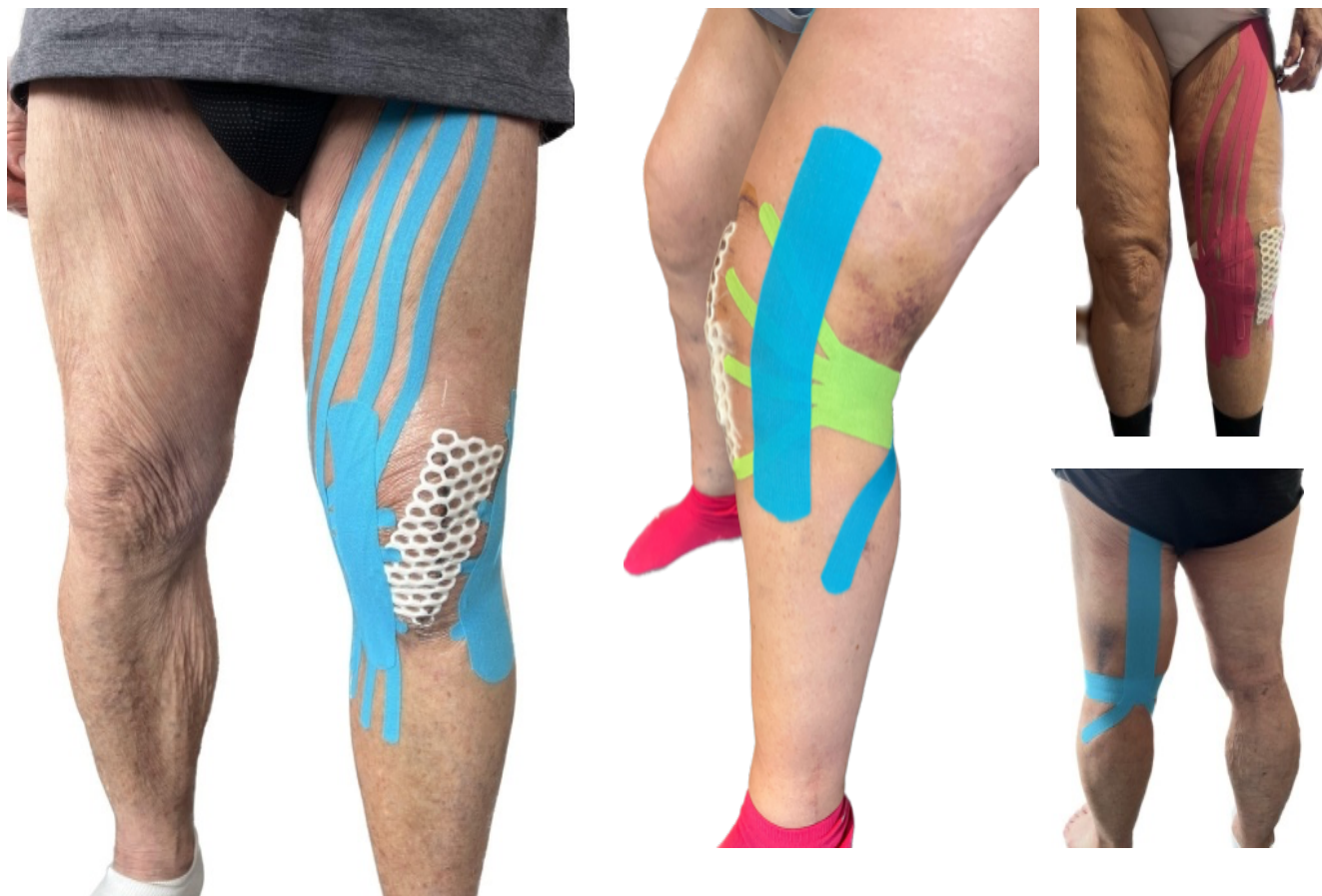

Supplement: Supplementary file 1 [file JRM-58-44964-s1.pdf]
